# Limited Predator-Induced Dispersal in Whiteflies

**DOI:** 10.1371/journal.pone.0045487

**Published:** 2012-09-27

**Authors:** Rui-Xia Meng, Maurice W. Sabelis, Arne Janssen

**Affiliations:** 1 Laboratory of Entomology, Inner Mongolia Agricultural University, Huhhot, People’s Republic of China; 2 Department of Entomology, China Agricultural University, Beijing, People’s Republic of China; 3 IBED, Population Biology, University of Amsterdam, Amsterdam, The Netherlands; University of California, Berkeley, United States of America

## Abstract

Whereas prey are known to avoid habitats with their predators, it is less well established whether they are triggered to emigrate to new habitats when exposed to predators in their current habitat. We studied plant-to-plant dispersal of adult whiteflies in response to the presence of predatory mites on the plant on which the whiteflies were released. These predators attack whitefly eggs and crawlers, but not the adults, which can fly to other plants and can learn to avoid plants with predators. Being tiny and wingless, the predatory mites are slow dispersers compared to adult whiteflies. This offers the whiteflies the opportunity to escape from plants with predatory mites to plants without predators, thus avoiding predation of their offspring. To test for this escape response, a greenhouse experiment was carried out, where whiteflies were released on the first of a row of 5 cucumber plants, 0.6 m or 2 m apart, and predators either on the same plant, on the next plant, or nowhere (control). Adult whiteflies dispersed significantly faster from plants with predatory mites onto neighbouring plants when the plants were 0.6 m apart, but not when plants were 2 m apart. However, the final numbers of whiteflies that had successfully dispersed at the end of the experiments did not differ significantly for either of the two interplant distances. Overall, the proportion of whiteflies that did disperse was low, suggesting that adult whiteflies were apparently reluctant to disperse, even from plants with predators. Our results suggest that this reluctance increases with the distance between the plants, so most likely depends on the uncertainty to find a new plant. Thus, whiteflies do not always venture to fly even when they can easily bridge the distance to another plant.

## Introduction

Dispersal is the process that connects subpopulations, thereby gluing them into one spatially-structured population [1], [2], [3], [4]. It hinges on two decisions: to emigrate from one subpopulation and to immigrate into another. There is ample empirical knowledge on the latter decision, i.e. patch or habitat selection by dispersing animals [5], [6], [7]. For example, animals choose habitats based on the availability and quality of food [8], [9], [10]. Furthermore, dispersing prey may avoid habitats with predators [5], [11], [12], [13], for example, tree frogs avoid ovipositing in ponds containing predators [14]. Animals may also avoid habitats with conspecific and heterospecific competitors [15], [16], [17], [18].

Whereas the decision for emigration from a habitat is also affected by the quality and quantity of food present in the habitat [9], [19], much less is known of how it is affected by the presence of predators. Although predators are reported to induce movement in prey [5], [12], [20], [21], [22], [23], most studies concern movement within habitats [24]. The few studies concerning dispersal across habitats focussed on increased emigration from a habitat as a result of predator presence [24], [25] or at increased frequencies of dispersal morphs in the populations exposed to predators [26], [27], [28]. However, these studies did not assess whether the induced emigration indeed resulted in increased successful immigration into other habitats. Hence, despite its obvious importance for (meta)population dynamics, predator-induced dispersal to new habitats is only poorly studied [24]. Throughout this article, we will refer to this combination of emigration and immigration as successful dispersal. In particular, we will quantify the successful dispersal of prey from a habitat with or without predators to other habitats (*i.e.* emigration followed by immigration of another habitat). In addition, we study the how the presence of predators in the other habitats affects prey immigration.

We used an experimental system consisting of the tobacco whitefly *Bemisia tabaci* (strain B (Gennadius)) and its natural enemy, the predatory mite *Amblyseius swirskii* (Athias-Henriot) on cucumber plants in a greenhouse. Dispersal of whiteflies to neighbouring plants is mainly by flight of winged adults [29], which are good dispersers [29] and are capable of finding host plants in a greenhouse within a matter of hours [13], [30]. The predatory mites attack whitefly eggs and crawlers, whereas later instars and adults are invulnerable [31]. They are small (<1 mm), blind, wingless arthropods that disperse by walking or by being transported passively by air currents [32]. In the experiments carried out here, only ambulatory dispersal was possible because air currents were not sufficiently strong to transport mites [33]. Release-recapture experiments in a greenhouse showed that whiteflies disperse c. 25 times faster than the predatory mites [13], [34]. We therefore expected the whiteflies to be able to escape from their predators by dispersing faster and farther away. Adult whiteflies previously exposed to predators that were feeding on whitefly eggs and crawlers avoid settling on plants with predatory mites, whereas whiteflies without such experience do not [13], [35].

We used this experimental system to test how the presence of predators affects the successful dispersal of adult prey to other plants at various distances. We specifically tested whether more prey successfully dispersed when predators were present in their habitat, and whether prey dispersed sooner or farther away when predators were present. Moreover, we studied whether the dispersing adult prey avoided settling on plants with predators.

## Materials and Methods

### Cultures

Cucumber plants (*Cucumis sativa* L. var. Ventura RZ®, RijkZwaan, De Lier, The Netherlands) were grown from seeds in pots (2 l) with soil in a climate room (25°C; 16 h photoperiod) for 2.5–3 weeks before being used for cultures and experiments. *Bemisia tabaci* strain B (J. Fransen pers. comm.) was obtained from a culture on poinsettia from the Research Station for Floriculture in Aalsmeer, The Netherlands, in March 1995, and was reared in climate boxes (27°C; 16 h photoperiod) on cucumber plants. The whitefly is a worldwide pest that can cause significant crop yield loss [36], [37].

The predatory mite *A. swirskii* is a new control agent for the biological control of tobacco whitefly (*Bemisia tabaci*), greenhouse whitefly (*Trialeurodes vaporariorum*), Western flower thrips (*Frankliniella occidentalis*), broad mites (*Polyphagotarsonemus latus*), chilli thrips (*Scirtothrips dorsalis*) and possibly also tomato russet mite (*Aculops lycopersici*) [31], [38], [39], [40], [41], [42], [43], [44], [45]. In this experiment we used a strain (Kazaa) that was collected from plants with whiteflies. The predators were reared on plastic arenas (8×15 cm) placed on a wet sponge in a plastic tray containing water [39]. Strips of wet tissue were placed on the plastic arena along its periphery so that the predators could obtain water from it, and glue barriers were applied on the wet tissue to prevent escape and contamination. A piece of transparent plastic sheet (1–2 cm^2^), bent in the shape of a tent, was placed on each arena to provide shelter for the mites. A few fibres of cotton wool were put underneath the shelter to serve as a substrate for oviposition. Mites were fed cattail (*Typha latifolia* L.) pollen twice per week. The predatory mite cultures were maintained in a climate room (25°C, 60% RH).

### Experimental Set-up

The general set-up of the experiment was as follows: in a greenhouse, 200–250 adult female whiteflies, collected from the culture, were released on one cucumber plant (c. 3 weeks old) and were allowed to settle for one day (see Table 1 for numbers released). To enable successful dispersal, four plants of the same age were subsequently placed in a row at one side of the release plant, to which the whiteflies could disperse. We investigated the effect of the presence of predators on successful dispersal by comparing the numbers of adult whiteflies that emigrated from release plants with predators and immigrated onto the neighbouring plants with those that successfully dispersed from release plants without predators. For the treatment with predators, 90 adult female mites, collected from the culture, were released on the same plant as the whiteflies (Table 1). Predators could also disperse from the plants on which they were released to the other plants, but they had to walk down a plant, over the soil, down the plant pot, onto the table plus the reverse sequence to reach another plant. Thus, predatory mites had a much harder job to successfully disperse than the adult whiteflies.

**Table 1:** Summary of the release schedule of the experiments.

Distance^1^	Predators	N^2^	Release plant^3^		Neighbouring plant^4^	
			whiteflies^5^	predators^6^	whiteflies^5^	predators^6^+ pollen
0.6	no predators	2	200	0	0	0
0.6	at 0 m	3	200	90	0	0
2	no predators	4	250	0	0	0
2	at 0 m	4	250	90	0	0
2	at 2 m	4	250	0	0	90
2	at 0 and 2 m	4	250	90	0	90

1 Distance between neighbouring plants in m;

2 Number of replicates;

3 The first plant of the row of five;

4 The second plant;

5 Number of adult whiteflies released;

6 Number of adult female predatory mites released.

Whiteflies were always released on the first plant (at 0 m) of a row of 5 plants. Female predatory mites were released on the first, second, or both plants. No whiteflies or predators were released on the other 3 plants.

To study the effect of the interplant distance on successful dispersal, the distance between neighbouring plants was either 0.6 m or 2 m (Table 1). This distance can easily be covered by flying adult whiteflies within a few hours [13]. We furthermore assessed whether adult whiteflies from release plants with predators immigrated onto neighbouring plants further away from the release plants than whiteflies from release plants without predators. We also assessed the effect of the presence of predators on neighbouring plants on successful prey dispersal, but with interplant distances of 2 m only. This was done by releasing predators onto the nearest neighbour of the plant on which the whiteflies were released (Table 1). Because these plants contained no prey at the start of the experiment, pollen (25–30 mg) was supplied as alternative food [46] to maintain the predator population. The presence of pollen does not affect predation of whitefly crawlers by the predatory mites [31], and whiteflies cannot feed on this pollen. Pollen was dusted onto the 2^nd^ lowest leaf during the first week, and onto the 4^th^ leaf of the same plant 1 week later. We subsequently assessed whether whiteflies dispersing from the release plant, which harboured predators or not, would preferentially fly to other plants than the plant harbouring predators. There were 6 treatments in total (see Table 1 for details and numbers of replicates).

The experiments were carried out in two greenhouse compartments (9×6 m, conditions set at 25°C, 60% RH, 16–8 h photoperiod, recorded every 30 min. with Gemini Data Loggers UK). The compartments were separated by crop-free corridors (3.2 m wide) to prevent cross-contamination, and were equipped with gauzed windows to reduce immigration of insects. Each compartment had four tables, 1.5 meters apart. Owing to restrictions of space in the greenhouse, we could not carry out all replicates of all treatments at the same time. Instead, we performed replicates of each treatment at the same time (in two greenhouse compartments), and repeated this to avoid differential effects of season on the treatments. Two treatments were conducted simultaneously in each compartment, with one empty table between the replicates. To check whether whiteflies dispersed from one set of plants to the other, we placed a clean cucumber plant on the empty table. We never found any whiteflies on this plant, indicating that very few whiteflies dispersed from one set of plants to the other. Hence, replicates carried out at the same time can be considered as independent. The experiments were conducted from 20 September 2004 until 16 March 2005, each experiment lasted for four weeks. The experiments with different interplant distances (0.6 or 2 m) were carried out in different periods and could therefore not be compared directly, but were instead compared to their respective controls without predators. Each replicate was done with a new set of plants, whiteflies, mites and pollen. The configuration of the set-up was also changed between replicates; hence, half of the replicates were done with the release plant on the right-hand side of the table, the other half with the release plant on the left-hand side. After each replicate, the table was cleaned thoroughly to remove predators and prey. A few clean cucumber plants were introduced into the greenhouse to trap whiteflies that had remained behind. A new replicate was started only after these plants attracted no further whiteflies.

No specific permits were required for the described greenhouse studies. The greenhouse where the experiments were carried out are owned and managed by the University of Amsterdam. The study organisms are plant pests and natural enemies and not endangered.

### Data Collection and Analysis

Daily censuses of the numbers of adult whiteflies on each neighbouring plant were done in the first week and every 3 days for the three subsequent weeks. Each leaf of each plant was inspected for adult whiteflies. Because disturbing one whitefly often triggers escape in nearby whiteflies, we refrained from counting adult whiteflies on the release plants, which had high densities of whiteflies throughout the experiment. Such flushing of whiteflies did not occur on the other plants, which had much lower densities. Owing to their small size, counting the predatory mites on intact plants would involve substantial manipulation of the plants (i.e. turning over leaves) and the use of a strong light source. As this would also disturb whiteflies and result in increased dispersal, predators were counted only at the end of the experiment.

Twenty-two to twenty-three days after the start of the experiment, the next generation of adult prey began to emerge, and the number of whiteflies on the plants were thus the result of dispersal and reproduction. Therefore, the numbers of dispersing whiteflies were analysed until day 22 only. Predators have a much shorter generation time, hence, the number of predators increased during the experiments.

Data on the dispersal of whiteflies consisted of repeated measures of counts, which were transformed to ln(x+1). The effects of treatments on dispersal of whiteflies were compared using linear mixed effect models (LME) corrected for repeated measures (package nlme of R, [47]), with time, the position of the plant and treatment as factors. Differences in numbers of adult whiteflies dispersing due to the presence of predators would result in a significant effect of the treatment, whereas differential speed or distance of dispersal would result in a significant interaction of the treatment with time or with the distance dispersed respectively. Linear mixed effect models were simplified by removing non-significant interactions and factors, and contrasts between treatments were assessed by combining factor levels [48]. Models were checked for heteroscedasticity and normality of errors.

At the end of the experiments (28 days), all adult whiteflies were counted while collecting them from all plants, including the release plants, with an aspirator. Subsequently, all leaves from all plants were detached and all adult female predatory mites were counted under a binocular microscope in the laboratory. The stem of the plant and the petioles were also checked for predators.

The final numbers of predators and whiteflies on all plants at day 28 were analysed with a generalized linear model (GLM in [47]) with a quasi-Poisson error distribution to correct for overdispersion. Treatments were compared with a Tukey test using the package multcomp (in R). The numbers of whiteflies on the non-release plants at day 22, hence, before the appearance of the new generation, were analysed using the same test.

## Results

**Figure 1:**
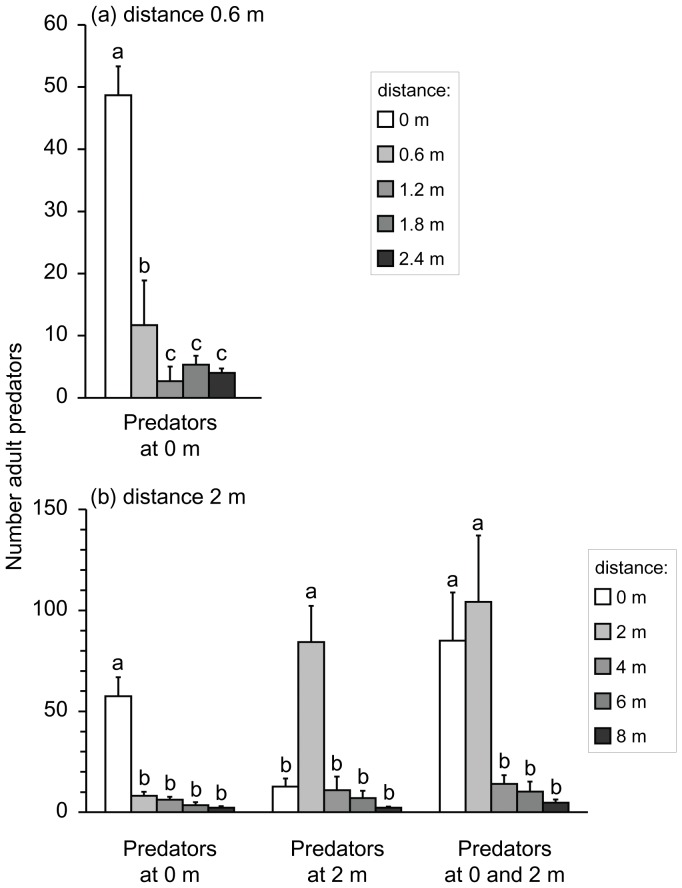
The average (+ s.e.m.) numbers of predatory mites (*A. swirskii*) on plants 28 days after they were released. (a) Distance between neighbouring plants 0.6 m. Predators were released on the plant at 0 m, on which whiteflies were also released. The average number of predators differed among plants (GLM, F_4,10_ = 14.7, P<0.001). (b) Distance between neighbouring plants 2 m. Predators were either released on the plant on which whiteflies were released at 0 m (Predators at 0 m), on the plant at 2 m (Predators at 2 m) or both on the plant at 0 and 2 m (Predators at 0 and 2 m). The average number of predators differed among plants in all three treatments (Predators at 0 m: F_4,15_ = 44.0, P<0.001; Predators at 2 m: F_4,15_ = 17.3, P<0.001; Predators at 0 and 2 m: F_4,15_ = 12.4, P<0.001). Within each treatment, bars with different letters differ significantly (contrasts after model simplification). See Table 1 for a summary of the release schedule.

After 28 days, some predatory mites had dispersed to plants on which they had not been released, but the numbers of predators on the plants on which they were released were significantly higher than on the other plants (Fig. 1). This confirms that the predators dispersed slowly [34], [49], and that our treatment successfully resulted in different numbers of predators on the plants. Hence, adult whiteflies were able to avoid predation of their offspring by dispersing from the release plant to plants with lower numbers of predators.

**Figure 2:**
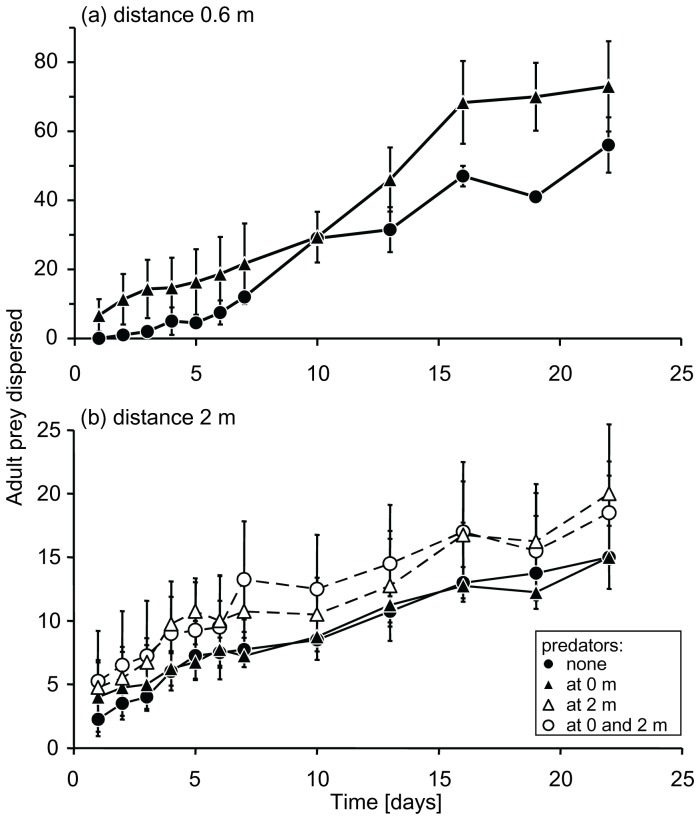
The average (± s.e.m.) numbers of adult whiteflies that dispersed from a release plant to 4 neighbouring plants through time. (a) Distance between neighbouring plants 0.6 m. Predators were either released on the same plant as the whiteflies (at 0 m) or no predators were released (none). (b) Distance between neighbouring plants 2 m. Predators were either released on the same plant as the whiteflies (at 0 m), on the plant closest to the release plant (hence, at 2 m), on the same plant as the whiteflies and on the neighbouring plant (at 0 and 2 m), or no predators were released (none). See legend to Figure 1 for further explanation.

**Figure 3:**
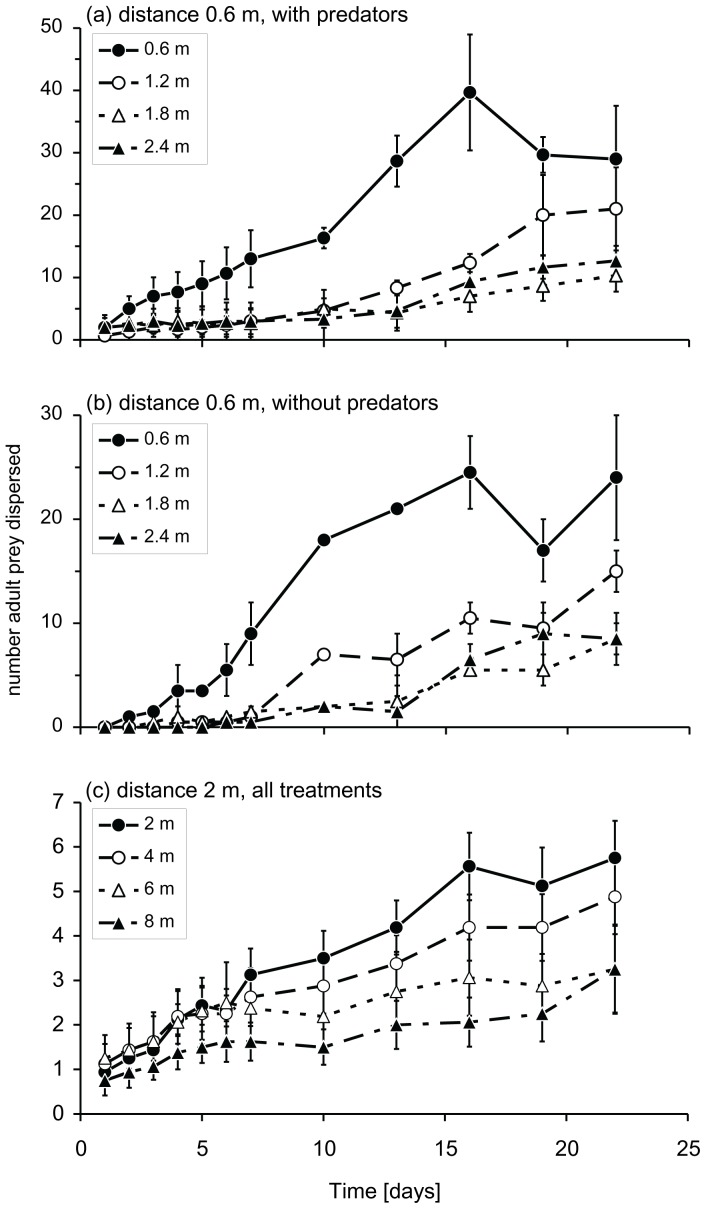
The average (± s.e.m.) numbers of adult whiteflies that dispersed to plants at various distances from the plant on which they were released as a function of time since release. (a) Distance between neighbouring plants 0.6 m, treatment with predators on the release plant. (b) Distance 0.6 m, treatment without predators. Significantly more prey were recaptured through time on the plant at 0.6 m. The two treatments are shown separately to demonstrate the absence of an interaction of treatment with the distance dispersed. (c) Distance between neighbouring plants 2 m. The numbers of prey recaptured did not differ significantly among plants.

With neighbouring plants at a distance of 0.6 m, the temporal pattern of successful dispersal from release plants with predators to neighbouring plants differed from that from release plants without predators (Fig. 2a: interaction of treatment with time, LME, L-ratio = 7.13, d.f. = 1, P = 0.0076). In particular, whiteflies dispersed more rapidly from plants with predators between 10–19 days (Fig. 2a). Overall, significantly more adult prey dispersed in the presence of predators on the release plant than in their absence (Fig. 2a: LME, Likelihood ratio = 13.6, d.f. = 1, P = 0.0002). However, the prey did not disperse farther away as a consequence of the presence of predators (interaction of treatment with the distance dispersed, LME, L-ratio = 3.43, d.f. = 3, P = 0.33, cf. Fig. 3a and b). Most prey dispersed to the plant closest to the release plant (Fig. 3ab).

With an interplant distance of 2 m, there was no effect of the presence of predators on successful dispersal of adult prey (Fig. 2b: L-ratio = 2.12, d.f. = 3, P = 0.55). Prey also did not disperse more rapidly or over larger distances as a result of the presence of predators (interaction between treatment and time: LME, L-ratio = 3.61, d.f. = 3, P = 0.31; treatment with position: L-ratio = 6.42, d.f. = 9, P = 0.70). There was a tendency of prey dispersing more to the plant closest to the release plant, but this trend was not significant (Fig. 3b).

Small differences in daily numbers of successful dispersers could potentially have led to large differences in numbers of dispersers over the entire experimental period. We therefore analysed the final numbers of whiteflies on the neighbouring plants separately (last data points in Fig. 2), but found no significant differences among treatments (GLM, 0.6 m: F_1,15_ = 1.98, P = 0.18; 2 m: F_3,57_ = 0.43, P = 0.73). Although we found significantly higher dispersal from plants with predators through time in the experiment with interplant distances of 0.6 m (Fig. 2a), this did not result in a higher number of adult whiteflies being found back on neighbouring plants at the end of the experiment. This shows that the presence of predators on the release plants resulted in a temporal shift in the dispersal (Fig. 2a).

**Figure 4:**
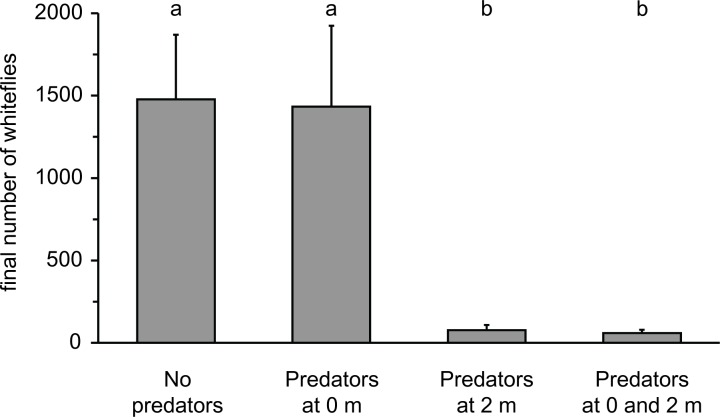
The average number of adult whiteflies (+ s.e.m.) on all five plants at the end of the experiment (after 28 days). Symbols above the bars indicate significant differences. Whitefly numbers were significantly higher in treatments in which predators were not released on the same plant as the whiteflies (left-hand two bars) than in treatments where predators and whiteflies were released on the same plant.

The total number of adult whiteflies present on all plants at the end of the experiment (28 d) gives an indication of the effect of the predators on the prey populations. With an interplant distance of 0.6 m, whitefly populations were significantly lower in the presence of predators than in their absence (average ± s.e.m. densities: without predators: 2148.5±959.5, with predators: 257. 7±90.1; GLM: F_1,3_ = 12.43, P = 0.039). With interplant distances of 2 m, the presence of predators on the release plant resulted in significant reductions of the whitefly populations (GLM: F_3,12_ = 14.18, P<0.001), but the presence of predators on the neighbouring plant had no significant effect (Fig. 4). These densities are relatively low: cucumber plants of similar size can easily harbour 5000–8000 adult whiteflies plus their offspring [40], [46], hence, competition for food cannot have played an important role during the experiments.

## Discussion

We found a significant temporal increase of successful prey dispersal (*i.e.* emigration from the release plant and immigration on one of the neighbouring plants) in the presence of predators when neighbouring plants were close by, not when neighbours were farther away (Fig. 2), showing that predatory mites can induce dispersal in adult whiteflies. When neighbouring plants were close by (0.6 m), some 28–36.5% of the 200 whiteflies dispersed successfully (Fig. 2a), most of them to the nearest plant (Fig. 3a). When alternative plants were farther away (2 m), 6–8% of the 250 adult whiteflies dispersed from the release plants to other plants. Thus, even when the predators induced significant successful dispersal in the whiteflies (with interplant distances of 0.6 m), a large majority (>>50%) of the whiteflies did not disperse. We therefore concentrate the remainder of the discussion on the general lack of dispersal of whiteflies in the presence of predators rather than emphasizing the significant but relatively small increase of successful dispersal in the presence of predators on the release plant.

Other studies have shown that whiteflies are good dispersers [29], and extremely efficient at finding new host plants [13], [35], [50]. Hence, the lack of successful dispersal observed here was not caused by limited dispersal capacity. A second explanation for the low numbers of successfully dispersing prey would be that the adult whiteflies cannot recognize the predators as being dangerous, because the adults are invulnerable to the predator used here [31]. However, adult whiteflies that have experienced predation on juveniles by *A. swirskii* are known to avoid settling in habitats with predators [13], [35]. Because the whiteflies on the release plants with predators did gain such experience, we expected that whiteflies would disperse from these plants.

Perhaps the limited dispersal of whiteflies compared to their potential to disperse [13], [35] points at an important aspect of dispersal of animals; successful dispersal depends on two decisions: the first being the decision to emigrate from habitat and the second the choice of a new habitat [51], [52]. Whereas dispersing whiteflies readily cover distances equal to those in the experiments reported here and avoid plants with predators [13], [35], the current study shows that they are reluctant to leave plants with predators. This reluctance may relate to the uncertainty to find a new habitat. With alternative habitats close by, dispersal would then be less risky than with habitats farther away, and this may explain the difference in dispersal with plants at different distances in the experiments described here. Possibly, the whiteflies were able to detect the neighbouring plants when they were 0.6 m apart, not when they were 2 m away, and consequently evaluated the risk of dispersal too high when plants were farther away.

Prey that did disperse were expected to avoid plants with predators [13], [35]. However, we found no evidence for this (Fig. 2b). We previously showed that adult whiteflies avoided plants with predators of which the past and present diet consisted of whitefly eggs and crawlers, but did not avoid plants with predators that fed on pollen [35]. The predatory mites on the plant neighbouring the release plant mainly fed on pollen in the beginning of the experiment. Hence, the lack of avoidance of this plant with predators may initially have been due to the whiteflies not responding to cues of pollen-fed predatory mites. In the course of the experiment, however, the predators on the plant neighbouring the release plant will have started to feed on the offspring of the adult whiteflies that settled and reproduced on this plant. However, the total numbers of whiteflies that settled on these plants were so low (Fig. 3c) that the numbers of eggs and crawlers may not have been sufficient food for the predators, so that their diet throughout the experiments will have consisted mainly of pollen.

We conclude that the predator-induced dispersal of whiteflies was predominantly to the nearest habitat (plant), and the tendency to disperse was affected to some extent by the presence of predators. Furthermore, the tendency to disperse was affected by the distance among habitats. Although there is substantial experimental work on the effect of predators or predator cues on habitat choice of prey [5], [11], [12], [53], there is considerably less known of predator-induced dispersal to predator-free habitats [24], [25], [26], [27], and even less of successful dispersal of prey from habitats with predators to safe habitats without these enemies. Our experiments suggest that the motivation to emigrate is more important for the distribution of animals over habitats than their capacity to cover the distances among habitats. It should be realized that predator-induced prey movement within a habitat is probably much less risky, and therefore more frequent, than predator-induced prey dispersal among habitats. Yet, it is successful dispersal across habitats that matters for the connectedness of local populations within a metapopulation.
